# Editorial: Understanding the Successful Coordination of Team Behavior

**DOI:** 10.3389/fpsyg.2017.01869

**Published:** 2017-10-27

**Authors:** Silvan Steiner, Roland Seiler, Nancy J. Cooke

**Affiliations:** ^1^Institute of Sport Science, University of Bern, Bern, Switzerland; ^2^Human Systems Engineering, Arizona State University, Mesa, AZ, United States

**Keywords:** team work, team sports, group, decision making, performance

In many areas of human life, people perform in teams. These teams' performances depend, at least partly, on team members' abilities to coordinate their contributions effectively (e.g., Steiner, 1972; Kravitz and Martin, 1986). This includes the making of decisions and the regulation of behavior in reference to the framework provided by the social group- and task-context (Wieber et al., 2012). Given the high relevance of a deepened and integrated understanding about the mechanisms underlying coordinated team behavior, the aim of this research topic is to provide a platform for different theoretical and methodological approaches to researching, describing, and understanding coordinated team behavior in different task contexts.

The 11 contributions accepted for publication in this Research Topic demonstrate that the understanding of coordinated team behavior defines a broad area of research: The researched teams are manifold and include rowing teams, soccer teams, rope skipping teams, baseball teams, scientific research teams, teams operating unmanned aerial vehicles, dyads that visually track multiple objects, and more. The diversity of the paradigms and approaches employed in the contributing articles does not fall short of that of the researched teams. This diversity illustrates the many considerable aspects of team coordination and signifies that various approaches are necessary to enable insights into the mechanisms potentially underlying team coordination in different situations. Although the employed approaches do differ from each other, they unite in their goal of overcoming the challenges that are associated with research on team coordination. Among others, these challenges include the actual measurement of coordination and the often limited accessibility of the underlying processes. In the following, examples of how these challenges are tackled shall be given to provide a short introduction into this Research Topic.

To assess the coordination of baseball infielders, Gray et al. use a novel joint decision paradigm involving a dedicated scoring system based on expert ratings of team coordination. By experimentally manipulating the composition of teams, the effects of previous common experiences on joint decisions are tested. In another experimental approach, Wahn et al. operationalize team coordination by the object-tracking performance of dyads. In the employed task, performance scores increase the more efficiently the partners divide task demands. Wahn et al. test how sharing (receiving) information about co-actors' actions and the team score affects team performance. Additionally, they test for differences in the effectiveness of specific coordination strategies over time.

Three contributions engage in network analysis. Pina et al. measure team performance by discriminating between successful and unsuccessful offensive plays in association football. They use social network analyses to calculate variables describing a team's passing network and test the predictive value of these network variables for the successfulness of team performance. Li et al. consider co-authorships of published articles as an indicator of team knowledge creation. Social network analysis is used to calculate variables describing the co-author networks and to test relations between network variables and team knowledge creation. Ramos et al. assent to the contributions of social networks analysis to understanding team behavior. With the goal of further expanding the capabilities of this methodological approach, the authors evaluate the use of hypernetworks that simultaneously access cooperative and competitive interactions between teammates and adversaries across space and time and on various levels of analysis.

In a case study involving a newly assembled rowing crew, Feigean et al. use boat velocity as a performance measure. They describe changes of the crew performance after a 6-week training interval and explore to which extent practice induced team benefits are obtained through distinct individual adaptions of the rowing patterns.

Stevens and Galloway use EEG data of the members of performing teams to quantitating the teams' neurodynamic organizations. Individual EEG data linked to measures of social coordination during the evolution of performed tasks are transformed into symbolic information units about the team's neural organization and synchronization. The authors discuss the potential the results raise for developing quantitative models of team dynamics that enable comparisons across teams and tasks.

Gesbert et al. adopt a phenomenological approach to explore how soccer players' lived experiences are linked to the active regulation of team coordination during offensive transition situations. They present different collective regulation modes that result from the qualitative analyses of the athletes' phenomenological reports.

Reviewing empirical findings, Gorman et al. illustrate the use of viewing teams as dynamical systems for understanding the coordination principles underlying teamwork. They advocate a systems perspective on teamwork that is based on general coordination principles lying within the individuals and present a framework for understanding and modeling teams as dynamical systems.

Steiner et al. provide an integrative perspective on coordination in interactive sport teams and define a framework that considers the coexisting contributions of shared mental models, situation-specific (ecological) information and individuals' constructionist perspectives on current game situations to enabling team coordination.

Bowers et al.'s contribution is dedicated to team resilience. The concept is used to explain why and how teams are able to maintain performance levels when facing adversity in the form of specific stressors. The authors provide a theoretical model of team resilience as an emergent state at the group level.

The contributions to this Research Topic offer a multifaceted insight into current research on team coordination and team functioning. We hope that they inspire further research on the topic as much remains to be learned about the successful coordination of team behavior. The many areas of human life in which performance is delivered by teams adumbrates the large field of application that could benefit from a deepened understanding.

## Author contributions

SS, RS, and NC contributed to the ms and gave final approval of the version to be submitted.

### Conflict of interest statement

The authors declare that the research was conducted in the absence of any commercial or financial relationships that could be construed as a potential conflict of interest.
